# Supplementary figures and images for: Sex differences in coronary artery calcium progression: The Korea Initiatives on Coronary Artery Calcification (KOICA) registry
Source: PLoS One. 2021 Apr 8;16(4):e0248884. doi: 10.1371/journal.pone.0248884 (PMC8031433; doi:10.1371/journal.pone.0248884)

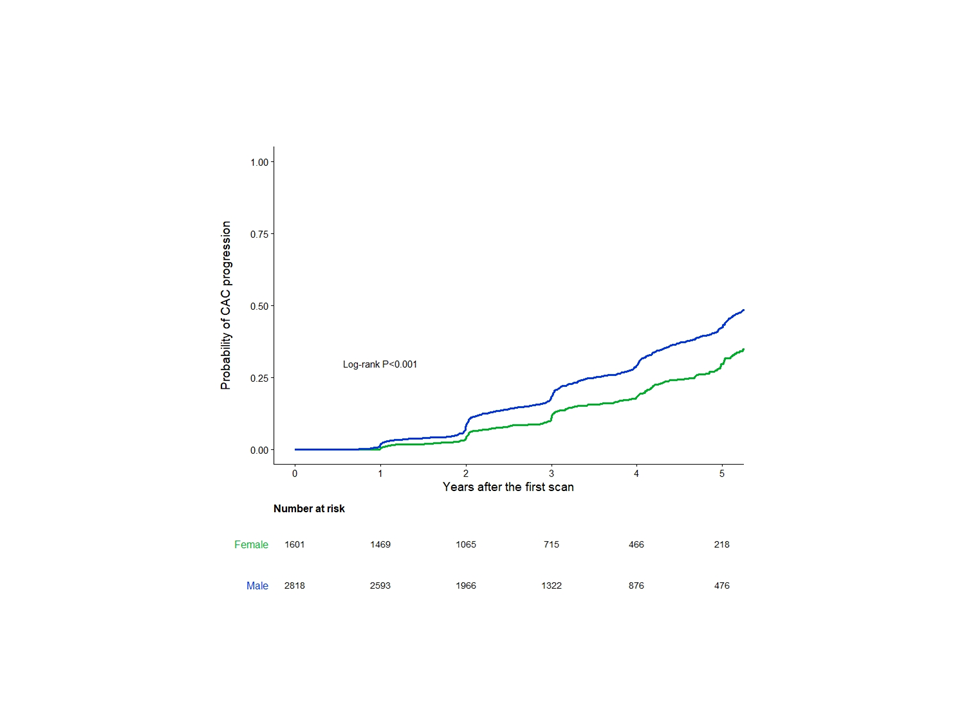

Supplement: S1 Fig — CAC, coronary artery calcium. (TIF) [file pone.0248884.s001.tif]

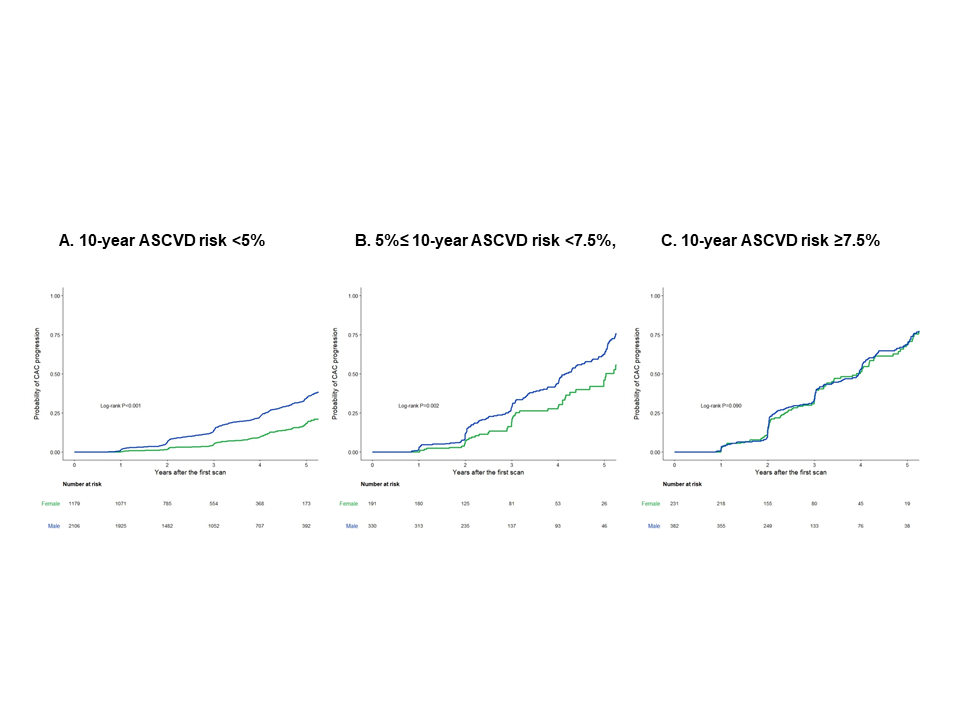

Supplement: S2 Fig — (A) 10-year ASCVD risk <5%, (B) 5%≤ 10-year ASCVD risk <7.5%, (C) 10-year ASCVD risk ≥7.5%. CAC, coronary artery calcium; ASCVD, atherosclerotic cardiovascular disease. (TIF) [file pone.0248884.s002.tif]
