# Supplementary material for: Sex differences in coronary artery calcium progression: The Korea Initiatives on Coronary Artery Calcification (KOICA) registry
Source: PLoS One. 2021 Apr 8;16(4):e0248884. doi: 10.1371/journal.pone.0248884 (PMC8031433; doi:10.1371/journal.pone.0248884)
Supplement: Attachment — Submitted filename: Review_Comments_to_the_Author_editing_final.docx [file pone.0248884.s003.docx]

**Journal Requirements**

**1. Please ensure that your manuscript meets PLOS ONE's style requirements, including those for file naming.**

**Re:** Thank you for your helpful comments. As requested, we have carefully rechecked the manuscript for compliance with the Journal’s requirements.

**2.  We note that you have indicated that data from this study are available upon request. PLOS only allows data to be available upon request if there are legal or ethical restrictions on sharing data publicly.**

**a) If there are ethical or legal restrictions on sharing a de-identified data set, please explain them in detail (e.g., data contain potentially identifying or sensitive patient information) and who has imposed them (e.g., an ethics committee). Please also provide contact information for a data access committee, ethics committee, or other institutional body to which data requests may be sent.**

**Re:** As previously described, the Korea Initiatives on Coronary Artery Calcification (KOICA) registry data cannot be made publicly available due to ethical restrictions set by the institutional review board committees of each participating healthcare center; i.e. public availability would compromise patient confidentiality and participant privacy. Therefore, access to aggregated data will be granted following review by the KOICA steering committee. The contact information for the committee is hjchang@yuhs.ac (+82-2-2228-8460, +82-10-4944-0423). This information has been added to the Data Availability Statement.

**3.  We noticed you have some minor occurrence of overlapping text with the following previous publication(s), which needs to be addressed: The text that needs to be addressed involves the Fig 2 caption, as well as sentences 3-5 of Page 19. In your revision ensure you cite all your sources (including your own works), and quote or rephrase any duplicated text outside the methods section. Further consideration is dependent on these concerns being addressed.**

**Re:** Thank you for pointing out this issue. Since we adopted the same definitions for CAC progression and CAC progression rate as those in JCCT 2017;11(5):347-53, we explained Figure 2 in a similar manner, resulting in overlap. Nevertheless, considering the concern regarding duplication, we have modified the legend for Figure 2 as follows:

**Page 11, lines 193-196: Fig 2.** **CAC progression for men versus women according to the 10-year ASCVD risk score. (A) Absolute difference in the CAC score between the first and last scanning sessions, (B) Annualized progression rate of CAC**

CAC, coronary artery calcium; ASCVD, atherosclerotic cardiovascular disease.

We apologize for the overlapping sentences in the limitations section (3-5 of page 19). We have revised the passage as follows:

**Page 21, lines 308-314:** Second, because of the absence of a specific study protocol guiding follow-up scanning, the interscan duration was relatively short [2.7 years (interquartile range, 1.9-4.1 years)] and was not constant. Nevertheless, the median duration between the initial and last scans did not differ between women and men. Furthermore, to minimize the potential influence of variations in the interscan duration, we analysed the association of annualized CAC progression with various cardiovascular risk factors, including male sex.

**Review Comments to the Author**

**Reviewer #1**

**This is an interesting study examining sex differences in CAC progression. The study has a number of strengths including large sample size of 9,675 men and 1,709 women with follow-up CAC scores. The authors did multivariate analysis as well as propensity matching to examine CAC progression rate in men vs women. An important finding is that among the high-risk groups by ASCVD score, that men and women had similar rates of CAC progression, and the male predominance of progression was only more pronounced in the lower risk groups. Overall I liked this paper and think the findings are important in adding to our understanding of CAC progression among men and women**

**1. Abstract – please state how progression of CAC is defined. As progression is likely more to occur among those with baseline disease, please also state prevalence of baseline CAC >0 among men and women, or median CAC score in abstract. Men might be more likely to progress is they have more disease to start with at baseline.**

**Re:** Thank you for your valuable suggestion. As recommended, we have added the definition of CAC progression and the prevalence of baseline CAC>0 at baseline to the abstract as follows:

**Page 3, lines 53-56:** At baseline, men were more likely to have a CAC score >0 than were women (47.8% vs. 28.6%). The probability of CAC progression at 5 years, defined as [√CAC score (follow-up) - √CAC score (baseline)] ≥2.5, was 47.4% in men and 29.7% in women (*p*<0.001).

**2. Did you exclude people with known clinical ASCVD? Was this a population of asymptomatic individuals? I would assume so, as the 10-year risk score applies in primary prevention not secondary prevention, but it does not explicitly state that this was a population without known clinical ASCVD.**

**Re:** Thank you for your valuable comment. First, we would like to explain how Korean health check-up centres work. Once every two years, Koreans may undergo health check-ups that are fully covered by the national health insurance system; however, if they wish, they can undergo the health check-up at specialized health check-up centres that do not receive any insurance coverage. In the former case, CAC scoring is not included in the health check-up program, whereas, in the latter case, individuals can choose the tests performed, including CAC scoring, and the costs are borne by the patients or their employer. Of course, patients with prior ASCVD can visit health check-up centres in a self-referral setting. However, most health check-up centres do not recommend CAC scoring in patients with known or suspected ASCVD, referring such patients to specialists. Therefore, the KOICA registry, with participants recruited from six healthcare centres, comprises apparently healthy individuals who underwent CAC scanning as a part of a health check-up.

When we evaluated prior ASCVD based on the responses to self-administered questionnaires, 683 (6.0%) participants indicated that they were diagnosed with ASCVD. However, considering the real clinical situation in Korea, these self-reported ASCVDs usually do not refer to the presence of angiographically proven significant CAD or ischemic stroke diagnosed based on brain MRI, but rather refer to the experience of atypical chest pain, facial palsy, or numbness, etc. Furthermore, considering that most health check-up centres do not recommended CAC scoring for patients with clinically significant ASCVD, we did not exclude patients with self-reported ASCVD. Nevertheless, when we performed the primary statistical analyses after the exclusion of participants with self-reported ASCVD (n=683) and those who did not indicate whether or not they had prior ASCVD (n=292), the results were not significantly changed. The figures corresponding to Fig 3 and Fig 4-C in the manuscript file, as well as the table corresponding to Table 3, are shown below. Therefore, we consider our decision to include these individuals to be reasonable.

**Response Letter Figure 1 (corresponding to Fig 3 in the manuscript). Kaplan-Meier 5-year CAC progression for men versus women in asymptomatic Korean individuals**

**Response Letter Figure 2 (corresponding to Fig 4A-C in the manuscript). Kaplan-Meier 5-year CAC progression for men versus women according to the 10-year ASCVD risk score. (A) 10-year ASCVD risk <5%, (B) 5%≤ 10-year ASCVD risk <7.5%, (C) 10-year ASCVD risk ≥7.5%.**

**Response Letter Table 1 (corresponding to Table 3 in the manuscript). Multivariate analysis for factors associated with the CAC progression rate**

|  | Model 1^a^ | | | | |  | Model 2^b^ | | | | |
| --- | --- | --- | --- | --- | --- | --- | --- | --- | --- | --- | --- |
|  | β | 95% CI | | | P value |  | β | 95% CI | | | P value |
| Male sex | 0.413 | 0.203 | - | 0.623 | <0.001 |  | 0.321 | 0.115 | - | 0.527 | 0.002 |
| Age | 0.032 | 0.020 | - | 0.044 | <0.001 |  | 0.021 | 0.006 | - | 0.033 | <0.001 |
| Body mass index, kg/m^2^ |  |  |  |  |  |  |  |  |  |  |  |
| Waist circumference, cm | 0.013 | 0.003 | - | 0.023 | 0.004 |  | 0.012 | 0.004 | - | 0.020 | 0.008 |
| Systolic blood pressure, mmHg |  |  |  |  |  |  |  |  |  |  |  |
| Diastolic blood pressure, mmHg |  |  |  |  |  |  |  |  |  |  |  |
| Hypertension | 0.440 | 0.297 | - | 0.583 | <0.001 |  | 0.349 | 0.206 | - | 0.492 | <0.001 |
| Diabetes | 0.396 | 0.110 | - | 0.682 | 0.006 |  | 0.366 | 0.086 | - | 0.646 | 0.01 |
| Dyslipidaemia | 0.307 | 0.164 | - | 0.450 | <0.001 |  | 0.269 | 0.128 | - | 0.410 | <0.001 |
| Current smoking | 0.294 | 0.157 | - | 0.431 | <0.001 |  | 0.283 | 0.148 | - | 0.418 | <0.001 |
| Haemoglobin, g/dL |  |  |  |  |  |  |  |  |  |  |  |
| Total cholesterol, mg/dL |  |  |  |  |  |  |  |  |  |  |  |
| HDL cholesterol, mg/dL | 0.004 | -0.002 | - | 0.010 | 0.125 |  |  |  |  |  |  |
| LDL cholesterol, mg/dL | 0.001 | -0.001 | - | 0.003 | 0.155 |  | 0.002 | 0.000 | - | 0.004 | 0.102 |
| Triglyceride, mg/dL | 0.001 | 0.001 | - | 0.001 | 0.002 |  | 0.001 | 0.001 | - | 0.001 | 0.005 |
| Creatinine, mg/dL | 0.367 | 0.020 | - | 0.714 | 0.038 |  | 0.393 | 0.050 | - | 0.736 | 0.025 |
| eGFR, ml/min/1.73 m^2^ |  |  |  |  |  |  |  |  |  |  |  |
| hs-CRP, mg/dL |  |  |  |  |  |  |  |  |  |  |  |
| Fasting glucose, mg/dL |  |  |  |  |  |  |  |  |  |  |  |
| HbA1C, % | 0.142 | 0.019 | - | 0.265 | 0.024 |  | 0.122 | 0.000 | - | 0.244 | 0.048 |
| 10-year ASCVD risk |  |  |  |  |  |  |  |  |  |  |  |
| Baseline CAC score |  |  |  |  |  |  | 0.004 | 0.004 | - | 0.004 | <0.001 |

HDL: high density lipoprotein; LDL: low density lipoprotein; eGFR: estimated glomerular filtration rate; hs-CRP: high-sensitive C-reactive protein; CAC: coronary artery calcium; ASCVD: atherosclerotic cardiovascular disease; HbA1c, glycated haemoglobin

^a^Model 1 adjusted for male sex, age, waist circumference, hypertension, diabetes, hyperlipidaemia, current smoking, LDL cholesterol, HDL cholesterol, triglyceride, creatinine, and HbA1c.

^b^Model 2 adjusted for the baseline CAC score in addition to the variables in Model 1.

**3. Time is a big risk factor for progression, need to account for time between CT scans. Individuals who had the 2 CTs close together will be less likely to have progression than if CTs were farther apart. The authors used annualized difference which I think is appropriate as it adjusts for time.**

**Re:** We share the reviewer’s concern regarding the potential effect of variations in the interscan duration. Although the interscan duration in men [median 2.8 years, (IQR 1.9-4.1)] was similar to that in women [median 2.6 years (IQR 1.9-4.1)], we took great care to minimize the effect of variations in the interscan duration. Specifically, we calculated the annualized CAC progression rate and evaluated the association between the annualized CAC progression rate and various cardiovascular risk factors, including male sex. This information has been added to the limitations section.

**Page 21, lines 314-321:** Furthermore, to minimize the potential influence of variations in the interscan duration, we analysed the association of annualized CAC progression with various cardiovascular risk factors, including male sex.

**4. I am also glad the adjusted for baseline CAC, because presence or absence of baseline CAC is a driver of CAC progression.**

**Re:** Thank you for pointing out this oversight. As you have pointed out, previous studies demonstrated that baseline CAC independently predicts CAC progression. (Atherosclerosis 2014;232:339–345). In the current study, there was a difference in baseline CAC score between men and women. Furthermore, baseline CAC was independently associated with the annualized CAC progression rate, as expected. Considering the effect of the baseline CAC score, we used two different multivariate models, with and without the baseline CAC score, to evaluate whether the association between male sex and CAC progression was affected by the baseline CAC score (Table 3). We have added footnotes to Table 3 to clarify the two models (see the response to #7).

**5. Can the authors add use of statins to Table 1? What was the use of statins in this population. Statin use has been shown to actually increase the CAC score despite its known risk reduction in CVD events (statins likely transform softer plaques into more stable dense plaques). The models should adjust for statin use.**

**Re:** We greatly appreciate your comment. We completely agree that it is very important to consider statin’s effect on CAC progression. However, unfortunately, as the questionnaire was self-administered, the collected medication history was incomplete; only 20% of study participants answered the question regarding statin use. Considering the low response rate reading medication usage, we did not include statin use in the analysis in the current study. Nevertheless, as this is an important issue, we have added the following sentence to the limitations section:

**Page 21, lines 318-322:** Finally, since we lacked detailed information regarding medication use, we could not include statin use in the multivariable analysis. Considering the emerging evidence suggesting that statins impact the increase in CAC, further studies are desired to evaluate whether the prominent CAC progression in men than in women is associated with greater statin use.

**6. Table 3 – change Male gender to Male sex. Sex is the more appropriate term here than gender since you are referring to likely biological differences related to sex hormones and other biological factors.**

**Re:** Thank you for your valuable comment. We agree with the reviewer and prefer “male sex” over “male gender”. We have modified Table 3 accordingly (see the response to comment #7).

**7. Also for table 3, include a footnote about what Model 1 and Model 1 adjusted for.**

**Re:** Thank you for your suggestion. We have added footnotes as follows:

Table 3. Multivariate analysis for factors associated with the CAC progression rate

|  | Model 1^a^ | | | | |  | Model 2^b^ | | | | |
| --- | --- | --- | --- | --- | --- | --- | --- | --- | --- | --- | --- |
|  | Β | 95% CI | | | P value |  | β | 95% CI | | | P value |
| Male sex | 0.396 | 0.186 | - | 0.606 | <0.001 |  | 0.270 | 0.079 | - | 0.459 | <0.001 |
| Age | 0.032 | 0.026 | - | 0.038 | <0.001 |  | 0.020 | 0.014 | - | 0.026 | <0.001 |
| Body mass index, kg/m^2^ |  |  |  |  |  |  |  |  |  |  |  |
| Waist circumference, cm | 0.012 | 0.006 | - | 0.018 | 0.002 |  | 0.010 | 0.004 | - | 0.016 | 0.006 |
| Systolic blood pressure, mmHg |  |  |  |  |  |  |  |  |  |  |  |
| Diastolic blood pressure, mmHg |  |  |  |  |  |  |  |  |  |  |  |
| Hypertension | 0.321 | 0.211 | - | 0.431 | <0.001 |  | 0.253 | 0.143 | - | 0.363 | <0.001 |
| Diabetes | 0.452 | 0.266 | - | 0.638 | <0.001 |  | 0.368 | 0.184 | - | 0.552 | <0.001 |
| Dyslipidaemia | 0.256 | 0.138 | - | 0.374 | <0.001 |  | 0.254 | 0.138 | - | 0.370 | <0.001 |
| Current smoking | 0.239 | 0.123 | - | 0.355 | <0.001 |  | 0.225 | 0.109 | - | 0.341 | <0.001 |
| Haemoglobin, g/dL |  |  |  |  |  |  |  |  |  |  |  |
| Total cholesterol, mg/dL |  |  |  |  |  |  |  |  |  |  |  |
| HDL cholesterol, mg/dL | 0.006 | 0.002 | - | 0.010 | 0.009 |  | 0.006 | 0.002 | - | 0.010 | 0.01 |
| LDL cholesterol, mg/dL | 0.002 | 0.000 | - | 0.004 | 0.044 |  | 0.002 | 0.000 | - | 0.004 | 0.025 |
| Triglyceride, mg/dL | 0.001 | 0.001 | - | 0.001 | <0.001 |  | 0.001 | 0.001 | - | 0.001 | <0.001 |
| Creatinine, mg/dL | 0.367 | 0.020 | - | 0.714 | 0.038 |  | 0.393 | 0.050 | - | 0.736 | 0.025 |
| eGFR, ml/min/1.73 m^2^ |  |  |  |  |  |  |  |  |  |  |  |
| hs-CRP, mg/dL |  |  |  |  |  |  |  |  |  |  |  |
| Fasting glucose, mg/dL |  |  |  |  |  |  |  |  |  |  |  |
| HbA1C, % | 0.105 | 0.017 | - | 0.193 | 0.02 |  | 0.100 | 0.012 | - | 0.186 | 0.027 |
| 10-year ASCVD risk |  |  |  |  |  |  |  |  |  |  |  |
| Baseline CAC score |  |  |  |  |  |  | 0.002 | 0.002 | - | 0.002 | <0.001 |

HDL: high density lipoprotein; LDL: low density lipoprotein; eGFR: estimated glomerular filtration rate; hs-CRP: high-sensitive C-reactive protein; CAC: coronary artery calcium; ASCVD: atherosclerotic cardiovascular disease; HbA1c, glycated haemoglobin

^a^Model 1 adjusted for male sex, age, waist circumference, hypertension, diabetes, hyperlipidaemia, current smoking, LDL cholesterol, HDL cholesterol, triglyceride, creatinine, and HbA1c.

^b^Model 2 adjusted for the baseline CAC score in addition to the variables in Model 1.

**8. It is disappointing that there was no menopause data in this cohort, but this was appropriately acknowledged as limitation by the authors.**

**Re:** Regrettably, we lacked data regarding menopause and hormone replacement therapy. We completely agree with the reviewer that it would be valuable to compare the CAC progression between postmenopausal women and age-matched men. We will consider this point in further studies to determine whether postmenopausal women may behave similarly to men in terms of the development and progression of coronary atherosclerosis.

**9. A major limitation of the study design is that this is self-referral cohort not a population based study, so there is referral bias – but this was acknowledged by authors. Men might be more likely to be referred for a second CAC scan compared to men, and indeed**

**Re:** We agree with the reviewer that the present study cohort of self-referred healthy individuals may not be fully representative of the general population. In fact, among the study participants, the number of men was larger than that for women, with a ratio of 5.6:1. As you mentioned, men are more likely to get health check-ups in a self-referral setting, and thus are more likely to get second CT scan. Nevertheless, as we acknowledged in the limitations section, the current study is a large multicenter study and reflects the reality that women seek fewer medical services than do men in this self-referral setting.

**10. Another limitation that should be mentioned is the relatively short follow-up time between scans, and sex differences in CAC progression over a longer period (i.e >10 years) could not be examined but would be of interest.**

**Re:** Thank you for your valuable comment. We agree that examining the long-term trend in CAC progression, as well as sex differences in this trend, would be a very interesting topic. Therefore, we have added the following sentence to the limitations section:

**Page 21, lines 308-314:** Second, because of the absence of a specific study protocol guiding follow-up scanning, the interscan duration was relatively short [2.7 years (interquartile range, 1.9-4.1 years)] and was not constant. Nevertheless, the median duration between the initial and last scans did not differ between women and men. Furthermore, to minimize the potential influence of variations in the interscan duration, we analysed the association of annualized CAC progression with various cardiovascular risk factors, including male sex.

**11. This is likely beyond the scope of this paper- but perhaps for the next paper, I am interested in knowing whether CAC progression is associated with incident ASCVD events incremental to risk conferred by baseline CAC, and if so, whether that association differed by sex. Some studies but not all have shown that an elevated CAC score in women confers greater CVD risk than it does in men. So is CAC progression in women also associated with greater CVD risk than in men?**

**Re:** We appreciated the opportunity to discuss this interesting topic with you. To our knowledge, CAC progression is known to be associated with incident ASCVD events, even after adjustment for baseline CAC (JACC Cardiovascular Imaging 2016;9(12):1420-1429). However, as you mentioned, whether the association differs by sex was not evaluated. It would be noteworthy to investigate whether CAC progression in women has a similar impact on the risk of ASCVD to that in men. In fact, KOICA investigators are currently preparing an outcome study; thus, we hope that we will be able to answer your question in our next project.
